# Retrospective case-control study of the impact of dialysis on bowel preparation scores

**DOI:** 10.1055/a-2565-8022

**Published:** 2025-05-12

**Authors:** Sébastien Kindt, Michele Vanhooren, Pieter Jan Poortmans, Karlien François

**Affiliations:** 1 60201 Gastroenterology and Hepatology, UZ Brussel, Brussel, Belgium; 2 70493 Vrije Universiteit Brussel, Brussel, Belgium; 3 60201 Gastroenterology and Hepatology, Universitair Ziekenhuis Brussel, Brussels, Belgium; 4 60200 Gastroenterology and Hepatology, University Hospital Ghent, Ghent, Belgium; 5 60201 Nephrology and Arterial Hypertension, UZ Brussel, Brussel, Belgium

**Keywords:** Endoscopy Lower GI Tract, CRC screening, Quality and logistical aspects, Preparation, Performance and complications

## Abstract

**Background and study aims:**

Inadequate bowel preparation (BP) negatively affects diagnostic performance of colonoscopy. Most trials assessing adequacy of bowel preparation regimens have excluded patients affected by chronic kidney disease (CKD), especially patients on dialysis. This study aimed to assess the impact of dialysis on BP quality and adenoma detection rate (ADR) and identify factors related to quality of BP.

**Patients and methods:**

We retrospectively compared patient-specific, preparation-specific (preparation solution, preparation regimen (split-dose vs. 1-day preparation, outpatient preparation), and colonoscopy-specific data (indication, Boston Bowel Preparation Score [BBPS], sedation type, presence of adenoma or cancer) between 79 patients on dialysis and 158 matched controls. Adequate BP was defined as a BBPS score ≧2 in every colonic segment. Significant contributors to BP were assessed by logistic regression.

**Results:**

Despite matching, dialysis patients were significantly older (69.0 ± 11.9 vs 64.2 ± 14.6, *P* = 0.008) and less frequently women (30% vs 52%, *P* = 0.002). There was no significant difference in BP or ADR between patients on dialysis and controls (85% vs 89%, *P* = 0.39 and 35% vs 35%, *P* = 1.00, respectively). Older age ( *P* = 0.03), lower body mass index ( *P* = 0.03), type of BP regimen ( *P* <0.001), outpatient preparation ( *P* = 0.03), and residency in residential care ( *P* = 0.05) were significantly associated with BP adequacy. According to the logistic regression model, split-dose regimen was the main predictor of adequate BP ( *P* <0.001, odds ratio 3.1 [1.65–5.81]).

**Conclusions:**

Safe and adequate BP is achievable in dialysis patients. Bowel preparation regimen rather than treatment with dialysis influences BP quality. Split-dose preparation remains the most important determinant of adequate BP for colonoscopy, irrespective of regimen.

## Introduction

Inadequate bowel preparation (BP) negatively affects the diagnostic performance of colonoscopy. On an individual level, inadequate BP is associated with lower cecal intubation rates, repeat procedures, missed adenomas, and lower patient satisfaction 1 2 3 . At a societal level, it is associated with increased post-colonoscopy colorectal cancer rates and increased healthcare costs 1 2 3 . Therefore, the European Society of Gastrointestinal Endoscopy (ESGE) advocates a rate of adequate BP of minimally 90% as a performance measure for lower gastrointestinal endoscopy 4 . In addition, ESGE recommends assessment of BP quality by a validated scale. The Boston Bowel Preparation Scale (BBPS) has been validated extensively and is internationally adopted for this purpose 5 .

Multiple studies have investigated adequacy and safety of different BPs. In general, international recommendations favor a split-dose regimen over 1-day preparations to ensure sufficient BP 6 . Multiple factors impact BP 7 . These include non-modifiable risk factors such as age and some comorbidities (e.g. cirrhosis and diabetes mellitus), as well as modifiable risk factors such as type of BP solution, preparation regimen, prior low-fiber diet, concomitant medication (e.g. narcotics), and inpatient status 8 . Patients treated with dialysis frequently suffer from comorbidities such as diabetes mellitus and/or heart failure, hence BP is expected to be worse compared with the general population. However, most of the trials assessing adequacy of bowel preparation regimens excluded patients affected by chronic kidney disease (CKD), especially patients on dialysis. As a result, data on the performance of BP in CKD remain limited and existing evidence is at least conflicting 9 10 .

This study aimed to assess the impact of dialysis on BP quality and adenoma detection rate (ADR). A secondary aim was to identify factors related to quality of BP.

## Patients and methods

This retrospective case-control study was conducted at the Department of Gastroenterology and Hepatology of the University Hospital Brussels, Brussels, Belgium. Patients on dialysis undergoing a colonoscopy between January 2016 and December 2021 were retrospectively identified from medical records. The study was approved by the Ethics Committee of University Hospital Brussels. We excluded patients without recorded BBPS. Every included patient on dialysis was matched to two controls without CKD (eGFR > 60 mL/min/1.73m ^2^ , no dialysis) selected from the colonoscopy program. Matched controls underwent a colonoscopy within a 1-week period of the included case’s colonoscopy and were matched for closest age. When more than two controls fulfilled this criterion, controls of both sexes were selected if possible. The control selection timeframe was applied to minimize bias from the skill of the provider because the procedures were performed by both experienced endoscopists and endoscopists in training. When a subject underwent multiple colonoscopies during the study period, only data from the first colonoscopy were collected.

We retrieved data from medical records that were patient-specific (sex, age, body mass index [BMI], history of abdominal surgery, presence of diabetes mellitus, residency in residential care), preparation-specific (preparation solution, preparation regimen (split-dose vs. 1-day preparation, outpatient preparation), and colonoscopy-specific (indication, BBPS, type of sedation, presence of adenoma or cancer). Dialysis-specific data such as etiology of nephropathy, duration (in years) and modality of dialysis (hemodialysis vs peritoneal dialysis) were retrieved. Adequate BP was defined as a BBPS ≥ 6 with a score of 2 or more in every segment of the colon, as determined by international consensus 6 .

For the primary objectives BBPS, adequate BP and ADR were compared between both groups by *t* -test and Chi-square test as appropriate. For the secondary objectives, we compared frequencies of adequate BP in relation to different patient characteristics by Chi-square or Fischer exact test as appropriate. Variables with a significant relationship were included as predictors and adequate BP as outcome in a logistic regression model with backward stepwise selection to account for multiple comparisons. At each step variables were chosen based on *P* = 0.1 as the threshold.

All statistical analysis were performed in SPSS v29 (IBM corporation). Results from continuous data are reported as mean ± standard deviation or median with interquartile range (IQR) as appropriate. Categorical data are reported as percentages. *P* ≤ 0.05 was considered significant.

## Results

### Study population

**Fig. 1:**
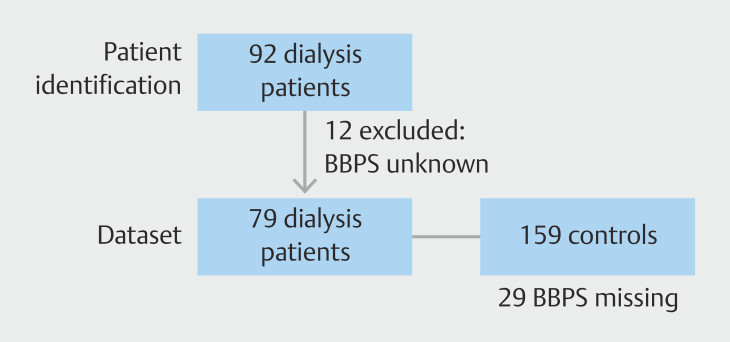
Flowchart of study subjects.

Ninety-one patients on dialysis underwent at least one colonoscopy during the study period. BBPS was missing for 12 patients, who were not included in the final dataset. Complete patient and colonoscopy data were available for 79 patients. These cases were controlled to 158 subjects without CKD. BBPS was missing for 29 controls. Fig. 1 is a flowchart of subject selection.

The majority of patients were men (69.6%) as compared with 48.1% of controls. Patients undergoing dialysis were statistically significantly older than controls (69.0 ± 11.9 vs. 64.2 ± 14.6, *P* = 0.008). BMI (26.3 ± 5.5 vs. 25.5 ± 5.4, *P* = 0.25) and BMI categories ( *P* = 0.66) did not differ between the two groups. Almost all subjects in both groups underwent colonoscopy for diagnostic purposes (73/79 vs. 150/158, *P* = 0.37). There was no difference in type of sedation between groups (midazolam: 55/79 vs. 108/158, propofol: 22/79 vs. 48/158, missing data: 2/79 vs. 2/158, for patients vs. controls resp., *P* = 0.76). Table 1 provides an overview and comparison of the characteristics of both groups.

**Table:** **Table 1** Comparison of study groups.

	Dialysis (n = 79)	Control (n = 158)	P value
Women (%)	24 (30%)	82 (52%)	0.002
Age (years)	69.0±11.9	64.2±14.6	0.008
BMI (kg/m ^2^ )	26.3±5.5	25.5±5.4	0.253
BMI category			0.656
<18.5 kg/m ^2^	3	10	
18.5–25 kg/m ^2^	30	68	
25–30 kg/m ^2^	26	45	
> 30 kg/m ^2^	20	34	
Non-interventional indication	73	150	0.369
Type of anesthesia			0.763
Midazolam	55	108	
Propofol	22	48	
Unknown	2	2	
BMI, body mass index.			

Patients underwent dialysis for a median of 2.0 years (IQR 0.0–4.0). Diabetic nephropathy was the leading cause of kidney failure in 28 patients, closely followed by vascular kidney disease in 24 patients. Twenty-three patients were listed for transplantation. Sixty-nine patients were on hemodialysis, and 10 received peritoneal dialysis. Table 2 provides an overview of the characteristics of the dialysis patients.

**Table:** **Table 2** Characteristics of patients on renal replacement therapy.

Total number of patients	79
Duration of dialysis (years)	2.0 (0.0–3.8)
Dialysis modality	
Hemodialysis	69
Peritoneal dialysis	10
Type of nephropathy	
Diabetic	28
Vascular kidney disease	24
Glomerulonephritis	9
ADPKD	5
Cristal nephropathy	2
Other	11
Patient on transplant list	23
ADPKD, autosomal dominant polycystic kidney disease.	

### Bowel preparation adequacy and ADR between groups

**Fig. 2:**
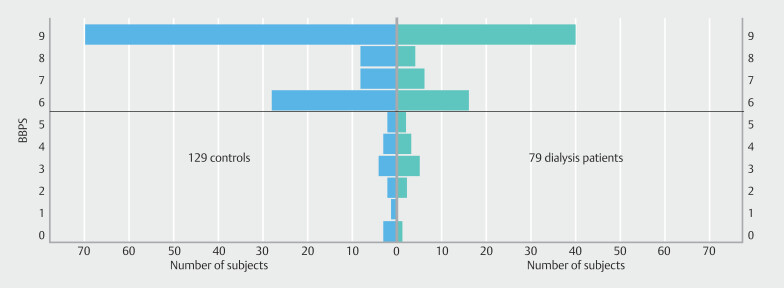
Distribution of BBPS between cases and controls.

BBPS overall was excellent and did not differ between patients on dialysis and controls (median 9 [6–9] vs. 9 [6–9], *P* = 0.95, mean 7.2 ± 2.2 vs. 7.4 ± 2.2). No difference was noted in proportion of adequate BP between patients and controls (85% vs. 89%, *P* = 0.39). Distribution of the BBPS between patients and matched controls is shown in Fig. 2 .

There were no differences in ADR between both groups (28; 35% vs. 55; 35%) in dialysis vs. controls respectively, *P* = 1.00).

### Determinants of adequate bowel preparation

As summarized in Table 3 , age (65.5 ± 14.4 vs. 70.7 ± 10.1 for adequate vs inadequate BP, *P* = 0.03), BMI (26.2 ± 5.5 vs. 24.2 ± 5.2 for adequate vs inadequate BP, *P* = 0.04), BP regimen (105/118 vs. 6/14 for split-dose vs. non-split dose, respectively, *P* < 0.001), outpatient preparation (103/124 vs. 77/82 for inpatient vs outpatient, respectively, *P* = 0.03) and residency in residential care (174/196 vs. 8/12, for non-residential care vs. residential care, *P* = 0.05) were significantly associated with BP adequacy.

**Table:** **Table 3** Determinants of adequate bowel preparation.

	Adequate BP % (ratio)	Inadequate BP % (ratio)	*P* value
Age (years) $	65.5 ± 14.4	70.7 ± 10.1	**0.03**
BMI (kg/m ^2^ ) $	26.2 ± 5.5	24.2 ± 5.2	**0.04**
Type of BP regimen $			**< 0.001**
Split-dose	89.0% (105/118)	11.0% (13/118)	
Non-split dose	42.8% (6/14)	57.2% (8/14)	
Outpatient preparation $			**0.03**
Inpatient	83.1% (103/124)	16.9% (21/124)	
Outpatient	94.0% (77/82)	6% (5/82)	
Residential care resident $			**0.05**
No	88.8% (174/196)	11.2% (22/196)	
Yes	66.7% (8/12)	33.3% (4/12)	
Type of anesthesia used during colonoscopy			0.81
Midazolam	86.5% (128/148)	13.5% (20/128)	
Propofol	89.3% (50/56)	10.7% (6/56)	
Indication for colonoscopy			0.37
Interventional	100% (12/12)	0% (0/12)	
Diagnostic	87.2% (170/195)	12.8% (25/195)	
Adenoma/carcinoma found during colonoscopy			0.83
Absent	86.7% (118/136)	13.3% (18/136)	
Present	88.9% (64/72)	11.1% (8/72)	
Dialysis			0.39
Absent	89.1% (115/129)	10.9% (14/129)	
Present	84.8% (67/79)	15.2% (12/79)	
Prior abdominal surgery			0.33
Absent	86.0% (135/157)	14.0% (22/157)	
Present	92.2% (47/51)	17.8% (4/51)	
Cirrhosis			0.25
Absent	88.1% (171/194)	11.9% (23/194)	
Present	78.5% (11/14)	21.5% (3/14)	
Diabetes mellitus			0.26
Absent	89.4% (127/142)	10.6% (15/142)	
Present	83.3% (55/66)	16.7% (11/66)	
Age and BMI: Mean ± standard deviation depending on bowel preparation adequacy compared by *t* -test. Other variables: Frequencies depending on bowel preparation adequacy compared by Chi-square or Fischer exact test. $ variable included in logistic regression. BP, bowel preparation.			

In contrast, type of anesthesia (midazolam vs. propofol, *P* = 0.81), indication (therapeutic vs. diagnostic, *P* = 0.37), prior abdominal surgery (absent vs. present, *P* = 0.33), cirrhosis (absence vs. presence, *P* = 0.25), presence of adenoma or carcinoma (absent vs. present, *P* = 0.83), diabetes mellitus (absent vs. present, *P* = 0.26) and dialysis (absent vs. present, *P* = 0.39) did not significantly correlate with BP. Repeating the analysis segregating further into hemodialysis and peritoneal dialysis did not impact the results. According to the logistic regression model, split-dose regimen was the main predictor of adequate BP ( *P* <0.001, OR 3.1; 1.65–5.81). Results of backward stepwise logistic regression are presented in Table 4 .

**Table:** **Table 4** Result of backward stepwise logistic regression.

	Odds ratio	Confidence interval	*P* value
**Step 1**			
Age	1.01	0.98–1.05	0.47
BMI	0.98	0.89–1.07	0.59
Outpatient preparation	0.22	0.02–2.56	0.23
Residential care	1.56	0.28–8.67	0.61
Type of BP	3.77	1.80–7.90	<0.0001
**Step 2**			
Age	1.02	0.98–1.06	0.43
BMI	0.97	0.88–1.07	0.54
Outpatient preparation	0.22	0.02–2.56	0.23
Type of BP	3.67	1.76–7.63	0.001
**Step 3**			
Age	1.02	0.98–1.06	0.38
Outpatient preparation	0.20	0.02–2.30	0.20
Type of BP	3.85	1.87–7.92	<0.0001
**Step 4**			
Outpatient preparation	0.18	0.02–2.0	0.164
Type of BP	3.65	1.81–736	<0.0001
**Step 5**			
Type of BP	3.09	1.65–5.81	<0.001
Result of backward stepwise logistic regression with odds ratio and confidence interval at every step. BMI, body mass index; BP, bowel preparation.			

## Discussion

In our cohort, dialysis patients had similar rates of adequate BP compared with matched controls without CKD. No significant difference in ADR was observed between the two groups. Overall, split-dose preparation was the main determinant of adequate BP in our cohort.

The stringent definition of adequate BP as a BPPS subscore of at least 2 in each segment represents the major strength of this study. Indeed, Clark et al. demonstrated that a subscore of 1 or less in any segment was associated with higher rates of missed adenomas larger than 5 mm, thus requiring early repeat endoscopy 11 . This criterion is generally accepted as the target for adequate BP 12 . In addition, our study assessed the impact of kidney failure and dialysis modality on BP quality. Finally, the results were compared with control subjects undergoing a colonoscopy during the same study period, instead of a historical cohort. This removes possible bias introduced by changes in clinical practice during the study period, e.g. modification in written instructions for BP, as well as endoscopist experience.

Current data on adequacy of BP in patients with kidney failure and dialysis are scarce and conflicting, hampering a meaningful sample size calculation. In a retrospective study investigating risk of colonic neoplasia, Saumoy et al. found significantly worse preparation scores in 70 patients on hemodialysis as compared with 70 age-matched controls 9 . Ohmiya et al., on the other hand, observed a 94% adequate BP (BBPS ≥6) with polyethylene glycol-electrolyte lavage solution plus ascorbic acid in a prospective uncontrolled study 10 in patients with CKD or kidney failure treated with hemodialysis. In our study, patients with kidney failure treated by either hemodialysis or peritoneal dialysis had similarly adequate BP when compared with the population of age- and sex-matched controls without CKD. These results support the data presented by Ohmiya et al. 10 , albeit with slightly lower rates of adequate BP. This difference could result from a different definition of adequate BP. Ohmiya et al. defined adequate BP as a BBPS ≥ 6, whereas we adopted the stricter definition requiring a subscore of ≥ 2 in every colon segment. In addition, our study investigated the potential implication of different parameters on adequacy of BP.

In the study by Saumoy et al. 9 prevalence of adenomatous polyps in patients on hemodialysis undergoing screening colonoscopy for kidney transplant evaluation was significantly higher than in the control population (54.3% vs. 32.9%). In contrast, whereas our study included patients both on hemodialysis and peritoneal dialysis, including screening as well as other indications for referral, we identified no differences in ADR between patients and controls (both groups 35%) with the ADR well above the minimum performance standard of 25% as proposed by ESGE 4 . The observed ADR in our study cohort aligns more with the result of a recent systematic review and meta-analysis by Madi et al. examining prevalence of colon polyps in patients undergoing screening for kidney transplant evaluation 13 . This study demonstrated an ADR of 33.4% in patients with kidney failure as opposed to only 23.9% in the control group. Strict adherence to screening guidelines and early referral for colonoscopy could explain the similar ADR in both patients and controls.

Some limitations need to be taken into consideration. First, despite the matching procedure, a significant 5-year age difference in disfavor of dialysis patients was identified. Although younger age was associated with higher BP success in the overall cohort in univariate analysis, this association was not found to be significant in logistic regression analysis. Moreover, we did not observe a difference in BP between the older dialysis patients and the younger controls.

Second, this study included only patients on dialysis with recorded BBPS. In 12 patients, BP was scored subjectively and without subscoring of colonic segments. Because images were not routinely recorded, retrospective assessment of BP was not possible. We excluded these patients because the subjective score could not guarantee adequate preparation in each segment of the colon, with subsequent uncertainty about the scoring of adequate BP. It cannot be excluded that this introduced selection bias. Concerning controls, BBPS was missing for 59 subjects. Nevertheless, further analysis was conducted including these controls, insofar as statistical methods did not require information about quality of BP.

Third, our results did not take into account withdrawal time because these data were not routinely collected, and thus, were unavailable for retrospective analysis. Withdrawal time represents an accepted performance measure of the quality of colonoscopic examination. Previous studies demonstrated a clear relationship between withdrawal time and ADR. In the current study, ADR did not differ between groups, suggesting comparable care in mucosal inspection during withdrawal.

Fourth, BP was adequate in slightly less than 90% of patients as well as controls. Several factors explain the failure to reach this target, as supported by literature 7 8 and our analysis. Inpatient preparation and residential care residents suffered a significantly lower preparation score. Older age, immobility, failure to follow a dietary regimen prior to colonoscopy, and associated medical conditions all impact BP adequacy. Also, inpatients are more likely to undergo a colonoscopy for urgent reasons, e.g. lower gastrointestinal tract bleeding, hampering strict adherence to BP guidelines.

Fifth, different endoscopists scored the BBPS, including both experienced endoscopists and those in training. Although BP evaluation by BBPS is part of the regular training, interobserver variation could affect the results, as demonstrated in earlier studies 5 . Mitigating this bias would require central reading of videotaped procedures. Sixth, some parameters known to influence BP were not systematically registered in medical records. This includes intake of drugs with anticholinergic properties or narcotics, habitual constipation as a comorbidity, or presence of heart failure. Finally, the retrospective nature of the study harbors a risk of selection bias and confounding. Indeed, the BP regimen depended on subject preference, whereas 1-day or split-dose was determined by timing of the colonoscopy, i.e. morning vs afternoon.

## Conclusions

In conclusion, safe and adequate BP can be achieved in dialysis patients. Our data show that BP regimen rather than presence of kidney failure and treatment by dialysis influences BP quality. Split-dose preparation remains the most important determinant of adequate BP for colonoscopy.
